# Clinical response and plasma levels of 5-fluorouracil in patients with colonic cancer treated by drug infusion.

**DOI:** 10.1038/bjc.1978.278

**Published:** 1978-12

**Authors:** B. L. Hillcoat, P. B. McCulloch, A. T. Figueredo, M. H. Ehsan, J. M. Rosenfeld

## Abstract

Concentrations of 5-fluorouracil (FU) were measured in the plasma of patients receiving i.v. infusions of the drug for 5 days as treatment for adenocarcinoma of the gastrointestinal tract. Concentrations of FU varied widely in many patients. Concentration of drug X time of infusion (C X t values) were calculated. Patients showing a partial response or stabilization of disease had significantly higher C X t values than non-responders. Methyl CCNU did not affect the C X t values of FU. Determination of the plasma concentration of FU would allow the dose of the drug to be adjusted to maintain high concentrations of FU in the plasma. Our data suggest that such high concentrations would increase the response rate in this disease.


*Br. J. Cancer* (1978) **38**, 719

### CLINICAL RESPONSE AND PLASMA LEVELS OF 5-FLUOROURACIL IN PATIENTS WITH COLONIC CANCER TREATED BY DRUG INFUSION

B. L. HILLCOAT*, P. B. McCULLOCH†, A. T. FIGUEREDO†, M. H. EHSAN* AND J. M. ROSENFELD‡

*From the *Department of Biochemistry, †Cancer Clinic and the ‡Department of Pathology, The Health Sciences Centre, McMaster University and the Cancer Clinic, Henderson Hospital, Hamilton, Ontario*

Received 21 June 1978 Accepted 31 August 1978

**Summary.—Concentrations of 5-fluorouracil (FU) were measured in the plasma of patients receiving i.v. infusions of the drug for 5 days as treatment for adenocarcinoma of the gastrointestinal tract. Concentrations of FU varied widely in many patients. Concentration of drug $\times$ time of infusion (C $\times$ t values) were calculated. Patients showing a partial response or stabilization of disease had significantly higher C $\times$ t values than non-responders. Methyl CCNU did not affect the C $\times$ t values of FU. Determination of the plasma concentration of FU would allow the dose of the drug to be adjusted to maintain high concentrations of FU in the plasma. Our data suggest that such high concentrations would increase the response rate in this disease.**

THE RESPONSE of adenocarcinoma of the gastrointestinal tract to treatment with FU is less when the drug is given orally than i.v. (Hahn *et al.*, 1975). Also, the various regimens used for i.v. treatment produce different response rates (Ansfield *et al.*, 1977). Of these regimens, continuous infusion of drug for 5 days is the least toxic (Seifert *et al.*, 1975) and, when combined with other drugs, gives good clinical responses (Woolley *et al.*, 1976). Some data are available on the plasma concentrations of FU after i.v. injection or oral administration (Cohen *et al.*, 1974), but only one other published study (Clarkson *et al.*, 1964) besides our preliminary report (Kawai *et al.*, 1977) gives data on plasma concentrations of drug during continuous i.v. infusion. These concentrations varied widely in the same patient during infusion, and the values of drug concentration $\times$ time varied widely between different patients. The present study was carried out to determine whether these drug concentrations related to tumour response and whether the administration of methyl CCNU on Day 1 of the infusion altered the plasma concentrations of FU.

### MATERIALS AND METHODS

*Selection of patients and treatment schedule.*—Patients with measurable metastatic adenocarcinoma of the gastrointestinal tract who had not received FU or methyl CCNU previously were treated by i.v. infusion of FU, 1·2 g/m$^2$/day (not $>$ 2 g) in 1 l of 5% dextrose for 5 days. Infusion was either by gravity or with a Holter pump, on a non-random basis. Infusions were routinely supervised in a general medical ward. Some patients received methyl CCNU 150 mg/m$^2$ by mouth on Day 1. Treatments were repeated at intervals of 6 weeks, unless severe drug toxicity or progression of disease occurred. Plasma levels of FU were determined during one or more of these courses.

*Evaluation of toxicity and tumour response.*—Stomatitis was graded as 1 if present but

Address for reprints: Dr Brian L. Hillcoat, Laboratory of Medicinal Chemistry and Biology, Developmental Therapeutics Program, Division of Cancer Treatment, National Cancer Institute, Bethesda, Maryland 20014.

not affecting food intake, 2 if preventing intake of solid but not soft and liquid food, and 3 if preventing intake of anything by mouth. Haematological toxicity was not evaluated, as the patients were discharged from hospital at the time a fall in white cells and platelets would have occurred. Moreover, this form of toxicity is infrequent and asymptomatic when FU is given by continuous infusion at the dosage used (Seifert *et al.*, 1975).

A complete response was defined as the disappearance of all disease; partial response was the objective response as described by Seifert *et al.* (1975); stabilization of disease was defined as no increase in the size of measurable lesions and no appearance of new lesions and no deterioration in laboratory tests over a period of 60 days, or cases which did not fulfil the requirements for a partial response; progression of disease occurred if there was objective evidence of progression, seen as an increase in the size of metastatic lesions or the appearance of new lesions or increasingly abnormal values in laboratory tests.

*Determination of plasma levels of FU*.—Five ml of blood was taken from Patients 5, 6, 10 and 11 from 8 to 11 times, at varying intervals as shown in Table I. For the other patients, blood samples were removed at daily intervals. The blood removed by venepuncture was collected in EDTA and the plasma removed and frozen. Batches of plasma were thawed and extracted as described and the FU determined by a mass-spectrometric method, previously reported. The standard error of our method with repeated analyses on the same sample was $\pm 4\%$ and the sensitivity 5 ng/ml (Hillcoat *et al.*, 1976).

### RESULTS

Plasma concentrations of 5-fluorouracil varied considerably between patients and in the same patient during infusion. Typical data are shown in Fig. 1, in which one patient (3) showed low levels of drug and wide variation (10-fold) and another patient (25) high levels of drug and little variation (2-fold). Tables I and

FIG. 1.—Variations in the plasma levels of 5-fluorouracil in two patients during treatment by 5-day infusion of the drug. ○———○, Patient 3; ●———●, Patient 25, (Table III).

TABLE I.—*Plasma concentrations of 5-fluorouracil (ng/ml FU) during 5-day infusions by gravity*

| Group A: Patient | | | | | | | | | | | | | Range[1] |
|---|---|---|---|---|---|---|---|---|---|---|---|---|---|
| 5(a) | Time (h) | 8 | 13 | 32 | 56 | 63 | 80 | 87 | 104 | 111 | | | |
| | Level | 200 | 28 | 80 | 85 | 108 | 43 | 158 | 82 | 133 | | | 7-fold |
| 5(b) | Time (h) | 8 | 25 | 42 | 49 | 66 | 73 | 90 | 120 | | | | |
| | Level | 94 | 20 | 44 | 42 | 214 | 150 | 28 | 68 | | | | 11-fold |
| 6 | Time (h) | 1 | 17 | 24 | 41 | 48 | 72 | 89 | 93 | 113 | 117 | | |
| | Level | 16 | 5 | 36 | 59 | 5 | 88 | 99 | 95 | 119 | 54 | | 24-fold |
| 10 | Time (h) | 2 | 20 | 26 | 44 | 50 | 68 | 74 | 92 | 98 | 116 | 122 | |
| | Level | 157 | <5 | 42 | <5 | <5 | 35 | 328 | 53 | 136 | 403 | 120 | 81-fold |
| 11 | Time (h) | 3 | 16 | 23 | 40 | 47 | 64 | 71 | 88 | 112 | | | |
| | Level | 30 | 66 | 88 | 112 | 93 | 49 | 23 | 494 | 81 | | | 21-fold |

| Group B: Patient | Day 1 | 2 | 3 | 4 | 5 | Range[1] |
|---|---|---|---|---|---|---|
| 2 | 44 | 45 | 54 | 50 | 58 | Constant |
| 3 | 46 | 21 | 125 | 13 | 46 | 10-fold |
| 4 | 56 | 55 | 148 | 36 | 34 | 4-fold |
| 7 | 100 | 46 | 104 | 303 | 132 | 7-fold |
| 8 | 180 | 140 | 100 | 60 | 130 | 3-fold |
| 9(b) | 192 | 106 | 72 | 106 | <5 | 38-fold |
| 12 | 64 | 270 | 61 | 65 | 127 | 4-fold |
| 14 | —[2] | 370 | 470 | 83 | 53 | 9-fold |
| 16 | 250 | —[2] | 70 | 370 | 210 | 5-fold |
| 18 | 258 | 400 | 42 | 100 | 467 | 11-fold |
| 19 | 172 | 150 | 155 | 583 | 185 | 4-fold |
| 20 | 246 | 162 | 349 | 106 | 559 | 5-fold |
| 21 | 191 | 248 | 134 | 517 | 224 | 4-fold |
| 22 | 384 | 282 | 169 | 369 | 170 | 2-fold |
| 24(a) | 171 | 215 | 166 | 748 | 284 | 5-fold |
| (b) | 342 | 361 | 887 | 143 | 365 | 6-fold |
| 25 | 440 | 415 | —[2] | 971 | 354 | 2-fold |
| 27 | 180 | 536 | 344 | 2216[3] 2000[3] | 180 | 12-fold |

[1] ratio of maximum to minimum concentration.

[2] not done.

[3] repeated on same sample.

II give the drug levels for patients treated by gravity and pump infusion respectively. C × t values were calculated from the area under curves such as those in Fig. 1. These values are shown in Table III. Patients 5, 9 and 24 had 2 infusions each, and the average of these values was used for statistical analyses. For a distribution plot, C × t values were grouped in ranges of 5 units: 0–5, 5–10 etc. Fig. 2 is a distribution of C × t values. For all 27 patients (Table III), the mean C × t value was 24·2 units (mg h FU/ml), the median, 15·9 u and the mode (Fig. 2), 10–15 u. Patients showing toxicity to FU had a mean C × t value of 19·3 u and a median value of 14·5 u; non-toxic patients had a mean of 27·6 u and a median value of 17·7 u. Patients showing a partial response or stabilization of disease had mean values of 36·1 u and a median value of 29·9 u, and non-responders a mean of 19·2 u and a median of 15·8 u. Inspection of the data (Fig. 2) also indicates that responding patients (PR and S) had higher C × t values than non-responding patients (NR), while toxic patients (T1, T2, T3) did not have high C × t values. Statistical analysis was carried out by not assuming a normal distribution of values, since Fig. 2 shows skewing on the right. Wilcoxon's rank-sum test (Dixon & Massey, 1969) was therefore used and gave a probability of 0·05 (2-tailed) that the difference between responders and non-responders occurred by chance. The

TABLE II.—*Plasma concentrations of FU(ng/ml) during 5-day infusion by pump*

| Patient | Day 1 | 2 | 3 | 4 | 5 | Range[1] |
|---|---|---|---|---|---|---|
| 1 | 40 | 49 | 40 | 16 | 9 | 5-fold |
| 9(a) | 108 | <5 | 182 | 100 | 264 | 53-fold |
| 13 | 218 | 115 | 92 | 115 | 204 | 2-fold |
| 15 | 134 | 134 | 128 | 228 | 332 | 2-fold |
| 17 | 172 | 144 | 314 | 230 | 295 | 2-fold |
| 23 | 230 | 216 | 144 | 320 | 1128 | 8-fold |
| 26 | 385 | 528 | 725 | 428 | 392 | 2-fold |

[1] ratio of maximum to minimum concentration.

difference in the C × t values of toxic and non-toxic patients and of those receiving either FU alone or FU with MeCCNU were not significant.

### DISCUSSION

The drug 5-fluorouracil remains the most effective single agent in chemotherapy of adenocarinoma of the gastrointestinal tract, and is used in combination chemotherapy for this disease. Nevertheless, we do not know the best method and schedule of administration. Some studies indicate that an i.v. loading dose of FU gives the best response (Ansfield *et al.*, 1977) while others suggest that i.v. infusion for 5 days with or without other drugs increases the frequency of response and may increase survival (Grillo-Lopez *et al.*, 1977; Buroker *et al.*, 1977). Objective responses obtained by the Eastern Cooperative Oncology Group were 6% at a dose of 7·5 mg/kg, 20% at 15 mg/kg and 25% at 20 mg/kg (Horton *et al.*, 1970). Increasing the dose of FU during a 24h infusion, repeated in 1 or 2 weeks, allows high doses of drug (up to 16 g/24 h infusion) to be given with regression and stasis of large refractory tumours (Spiers *et al.*, 1977). This dependence of response on dose suggests that response may correlate with the plasma concentration of the drug, in spite of the complex bio-

TABLE III.—*C × t values of FU (mg/h/ml), patient response and drug toxicity*

| Patient | C × t | Response[2] | Toxicity | Infusion pump | MeCCNU |
|---|---|---|---|---|---|
| 1 | 3·0 | NR | 2 | + | − |
| 2 | 5·4 | NR | 0 | − | + |
| 3 | 5·6 | NR | 3 | − | − |
| 4 | 7·8 | NR | 0 | − | + |
| 5 | 10·6, 8·4 (9·5)[1] | NR | 0 | − | − |
| 6 | 10·2 | NR | 0 | − | − |
| 7 | 12·6 | NR | 3 | − | − |
| 8 | 13·6 | NR | 3 | − | + |
| 9 | 11·6, 16·7 (14·2)[1] | NR | 0 | + | − |
| 10 | 14·3 | S | 2 | − | − |
| 11 | 14·5 | S | 1 | − | − |
| 12 | 15·6 | S | 0 | − | − |
| 13 | 15·8 | NR | 0 | + | − |
| 14 | 15·9 | NR | 0 | − | − |
| 15 | 23·4 | NR | 3 | + | − |
| 16 | 23·5 | NR | 0 | − | + |
| 17 | 24·6 | NR | 3 | + | + |
| 18 | 28·1 | NR | 0 | − | − |
| 19 | 29·2 | PR | 3 | − | − |
| 20 | 29·6 | NR | 0 | − | − |
| 21 | 30·6 | S | 1 | − | − |
| 22 | 32·4 | NR | 0 | − | − |
| 23 | 38·9 | NR | 0 | + | + |
| 24 | 36·5, 45·3 (41)[1] | S | 1 | − | + |
| 25 | 51·2 | NR | 0 | − | − |
| 26 | 55·2 | S | 0 | + | − |
| 27 | 88·4 | PR | 0 | − | − |

[1] average of 2 infusions.
[2] NR, no response; S, stabilization; PR, partial response.

FIG. 2.—Distribution of C × t values of each of 27 patients. NR, non-responder; S, stabilized disease; PR, partial responder; T1, T2, T3, increasing levels of drug toxicity. The C × t values were calculated as areas under curves such as those in Fig. 1.

chemical and kinetic steps involved in the ultimate action of FU on the tumour cell. In a similar way, high plasma concentrations of methotrexate after large doses of the drug produce responses in tumours resistant to lower plasma concentrations. Plasma levels of FU are also important when the drug is given with thymidine. Phase I studies have then shown marked elevation and prolongation of FU levels in the plasma, with increased marrow toxicity compared to FU alone (Vogel *et al.*, 1978). The enhanced tumour effect reported in animals given this combination (Martin *et al.*, 1978) may result from the high level of FU maintained over a considerable period of time. However, the action of the drug under these conditions may be qualitatively different from that when the drug is given alone (Nayak *et al.*, 1978).

Our results indicate a positive correlation between plasma levels of drug and tumour response. Gudauskas and Goldie (1978) have recently presented data showing a similar correlation and confirming the variability we observe. Since FU given as an infusion is less toxic to the marrow than when given as a single i.v. injection (Seifert *et al.*, 1975) successive treatments by infusion could use increasing doses as needed to maintain a level of drug at or above 36·1 C × t units, the mean value for responders in our series.

Methyl CCNU did not alter plasma concentrations of FU, so the reported synergism of this agent with FU (Moertel *et al.*, 1975) is not due to changed plasma concentrations of the latter drug.

Plasma concentrations of FU fluctuated more widely with gravity infusion than with pump infusion, as expected. These concentrations reflect the short half-life (an $\alpha$ phase of 12 min) of the drug in the plasma (Kirkwood & Frei, 1978).

Our data indicate that an increased rate of response may result if the plasma concentration of FU were used to adjust the dose of drug administered. Combined with the method of predicting marrow toxicity which we reported previously (Hillcoat *et al.*, 1977) this approach may allow an optimum and individualized use of FU.

This work was supported by IBM (Canada). Grateful acknowledgement is made to the daughter, son and other relatives of Mrs Adams in providing the infusion pump used in this study.

### REFERENCES

ANSFIELD, F., KLOTZ, J., NEALON, T., & 6 others (1977) A phase III study comparing the clinical utility of four regimens of 5-fluorouracil. *Cancer*, **39**, 34.

BUROKER, T., KIM, P. N., HEILBRUN, L. & VAITKEVICIUS, V. K. (1977) 5-FU infusion with mitomycin-C vs. 5-FU infusion with methyl-CCNU in the treatment of advanced colon cancer. *Proc. Am. Soc. Clin. Oncol.*, **18**, 271.

CLARKSON, B., O'CONNOR, A., WINSTON, L. & HUTCHISON, D. (1964) The physiologic disposition of 5-fluorouracil and 5-fluoro-2′-deoxyuridine in man. *Clin. Pharmacol. Ther.*, **5**, 581.

COHEN, J. L., IRWIN, L. E., MARSHALL, G. J., DARVEY, H. & BATEMAN, J. R. (1974) Clinical pharmacology of oral and intravenous 5-fluorouracil (NSC-19893). *Cancer Chemother. Rep.*, **58**, 723.

DIXON, W. J. & MASSEY, F. J., JR (1969) *Introduction to Statistical Analysis*, 3rd edition. New York: McGraw-Hill. p. 344.

GRILLO-LOPEZ, A. J., VELEZ-GARCIA, E. & ELLIOTT, A. (1977) Survival of patients with advanced gastro-intestinal cancer treated with 5-fluorouracil (5FU) drip. *Proc. Am. Soc. Clin. Oncology*, **18**, 331.

HAHN, R. G., MOERTEL, G. C., SCHUTT, A. J. & BRUCKER, H. W. (1975) A double-blind comparison of intensive course 5-fluorouracil by oral *vs.* intravenous route in the treatment of colorectal carcinoma. *Cancer*, **35**, 1031.

HILLCOAT, B. L., BANERJEE, M., MCCULLOCH, P. B. & WILLIAMS, C. K. O. (1977) Prediction of marrow toxicity in patients treated by intravenous infusion of 5-fluorouracil. *Eur. J. Cancer*, **13**, 81.

HILLCOAT, B. L., KAWAI, M., MCCULLOCH, P. B., ROSENFELD, J. & WILLIAMS, C. K. O. (1976) A sensitive assay of 5-fluorouracil in plasma by gas chromatography-mass spectrometry. *Br. J. Clin. Pharmacol.* **3**, 135.

HORTON, J., OLSON, K. B., SULLIVAN, J., REILLY, C., SCHNIDER, B. & Eastern Cooperative Oncology Group (1970) 5-Fluorouracil in cancer: an improved regimen. *Ann. Intern. Med.*, **73**, 897.

KAWAI, M., ROSENFELD, J., MCCULLOCH, P. & HILLCOAT, B. L. (1977) Blood levels of 5-fluorouracil during intravenous therapy. *Br. J. Cancer*, **36**, 346.

KIRKWOOD, J. M. & FREI, E. (1978) 5-Fluorouracil (FU) with thymidine (TdR); a Phase I study. *Proc. Am. Assoc. Cancer Res.*, **19**, 159.

MARTIN, D. S., STOLFI, R. L. & SPIEGELMANN, S. (1978) Striking augmentation of the *in vivo* anticancer activity of 5-fluorouracil (FU) by combination with pyrimidine nucleosides: an RNA effect. *Proc. Am. Assoc. Cancer Res.*, **19**, 221.

MOERTEL, C. G., SCHUTT, A. J., HAHN, R. G. & REITEMEIER, R. J. (1975) Therapy of advanced colorectal cancer with a combination of 5-fluorouracil, methyl-1,-3-cis (2-chloroethyl)-1-nitrosourea and vincristine. *J. Natl Cancer Inst.*, **54**, 69.

NAYAK, R., MARTIN, D., STOLFI, R., FURTH, J. & SPIEGELMAN, S. (1978) Pyrimidine nucleosides enhance the anti-cancer activity of FU and augment its incorporation into nuclear RNA. *Proc. Am. Assoc. Cancer Res.*, **19**, 63.

SEIFERT, P., BAKER, L. H., REED, M. L. & VAITKEVICIUS, V. K. (1975) Comparison of continuously infused 5-fluorouracil with bolus injection in treatment of patients with colorectal adenocarcinoma. *Cancer*, **36**, 123.

SPIERS, A., STRAUS, M., JANIS, M., POLACKWICH, R. & MOZDEN, P. (1977) High dose intravenous infusions of 5-fluorouracil for refractory tumors—the Hi-FU regimen. *Proc. Am. Soc. Clin. Oncol.*, **18**, 292.

VOGEL, S., PRESANT, C., RATKIN, G. & KLAHR, C. (1978) Phase I study of infusion 5-fluorouracil (5FU) plus thymidine (T). *Proc. Am. Assoc. Cancer Res.*, **19**, 232.

WOOLLEY, P. V., III, MACDONALD, J. S. & SCHEIN, P. S. (1976) Chemotherapy of colorectal carcinoma. *Semin. Oncol.*, **3**, 415.


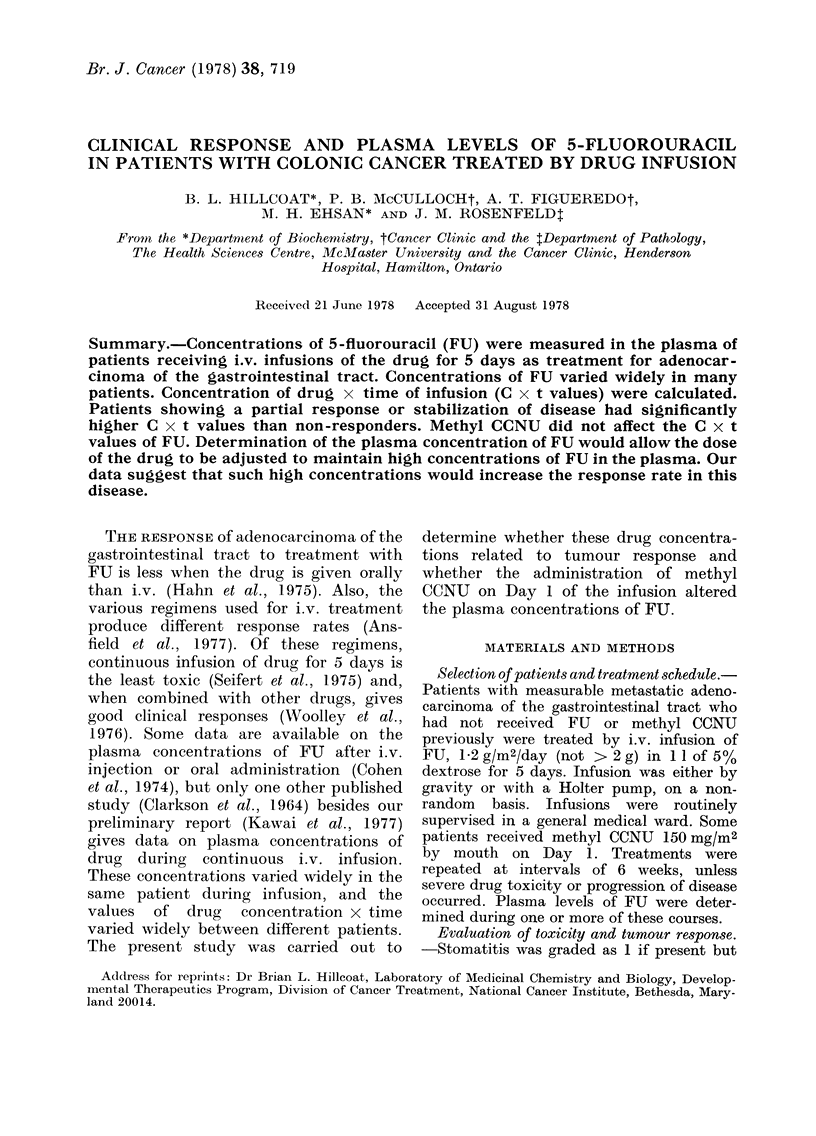

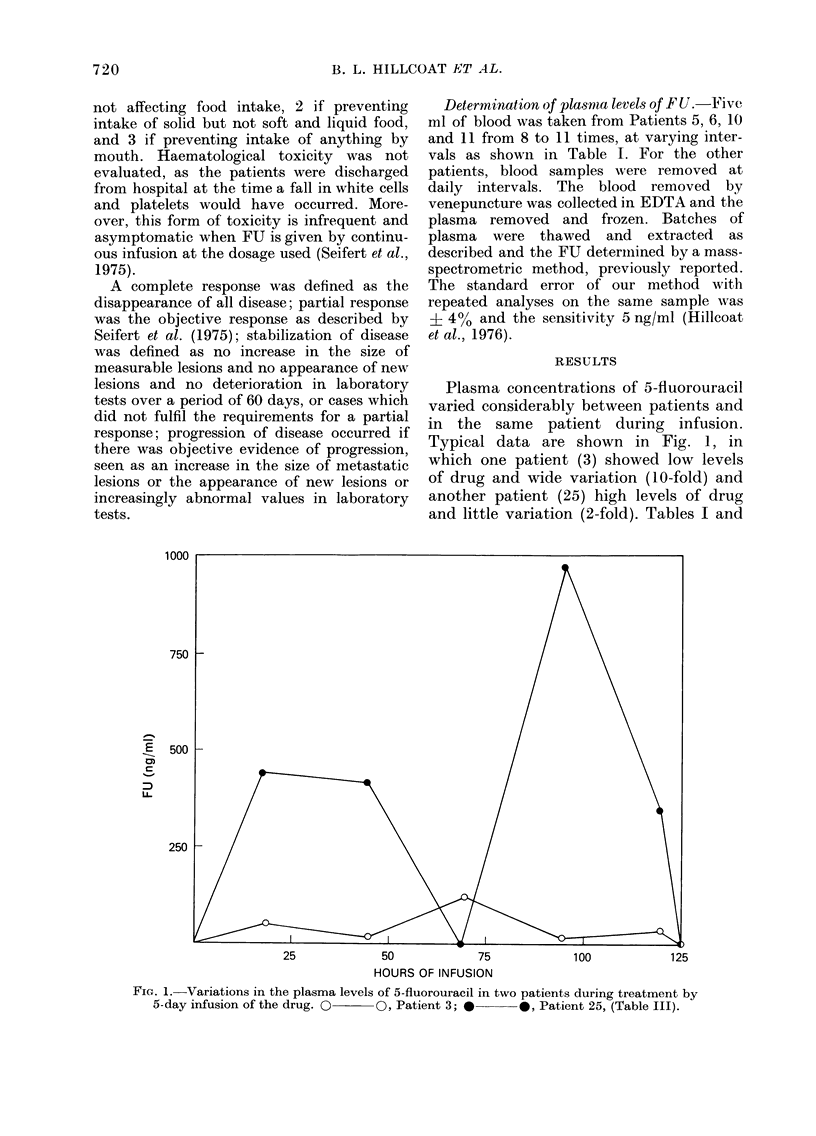

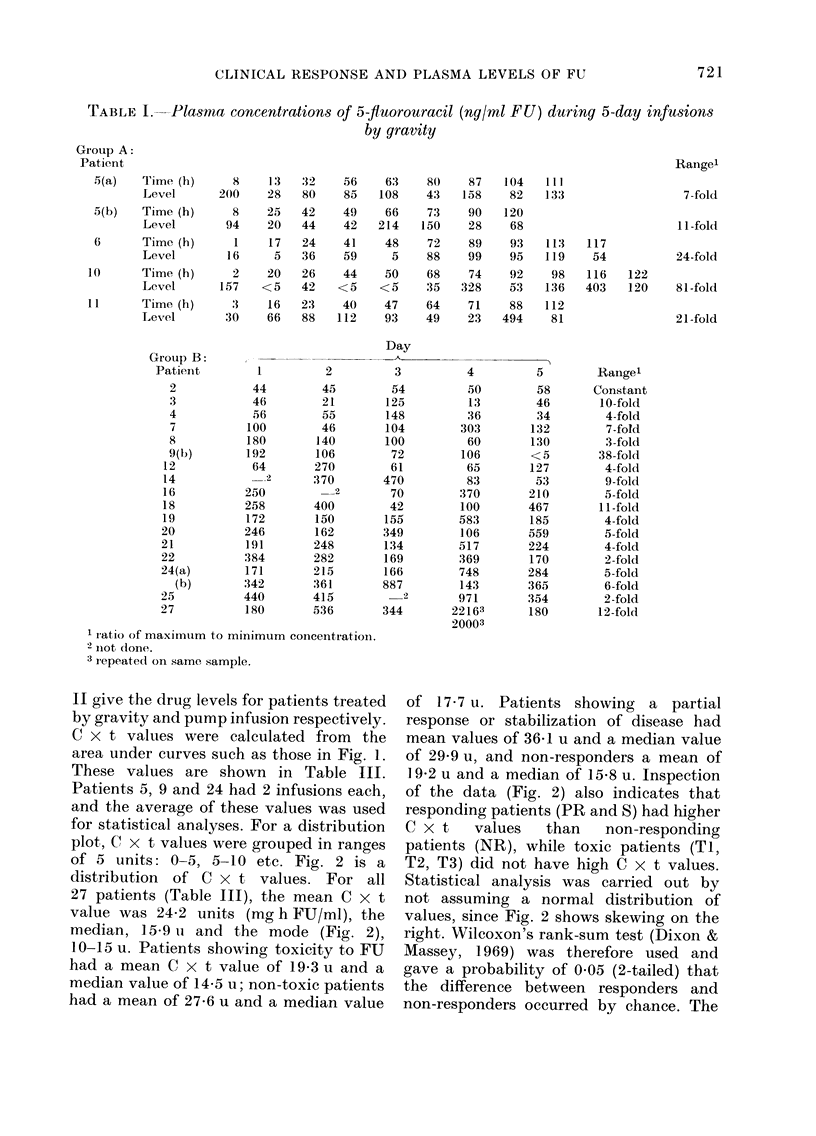

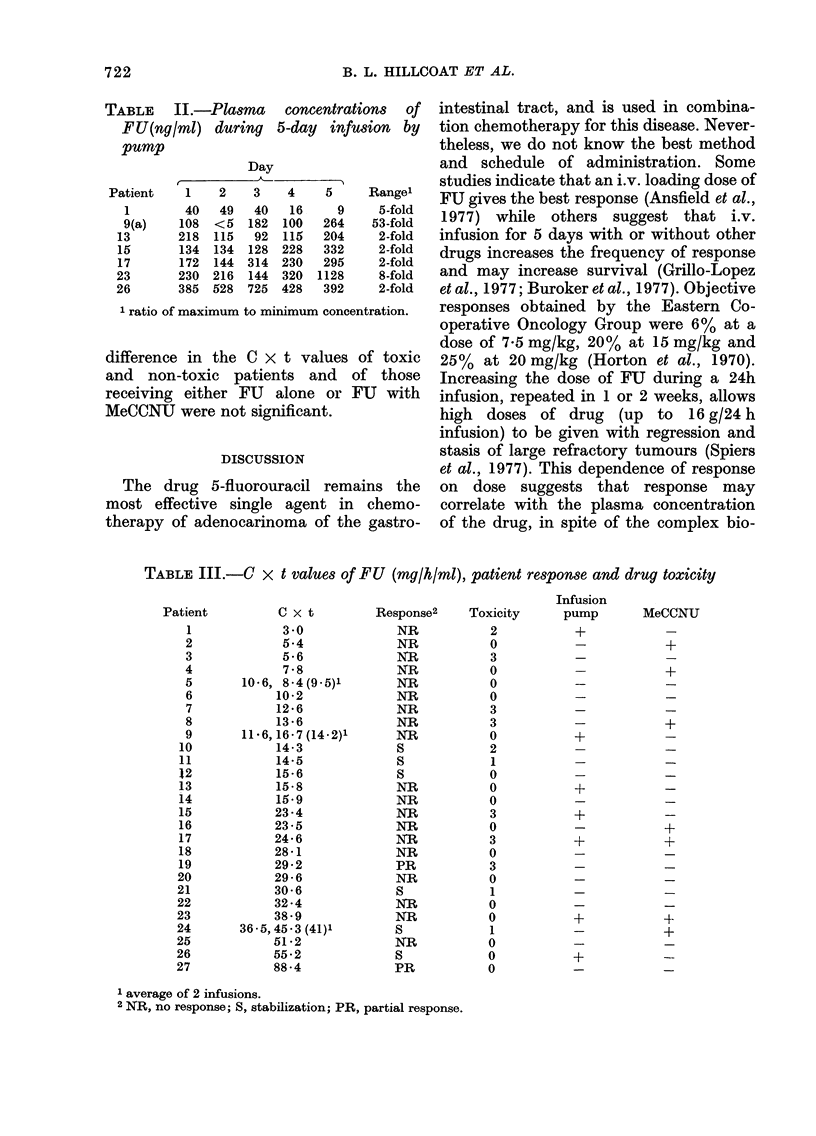

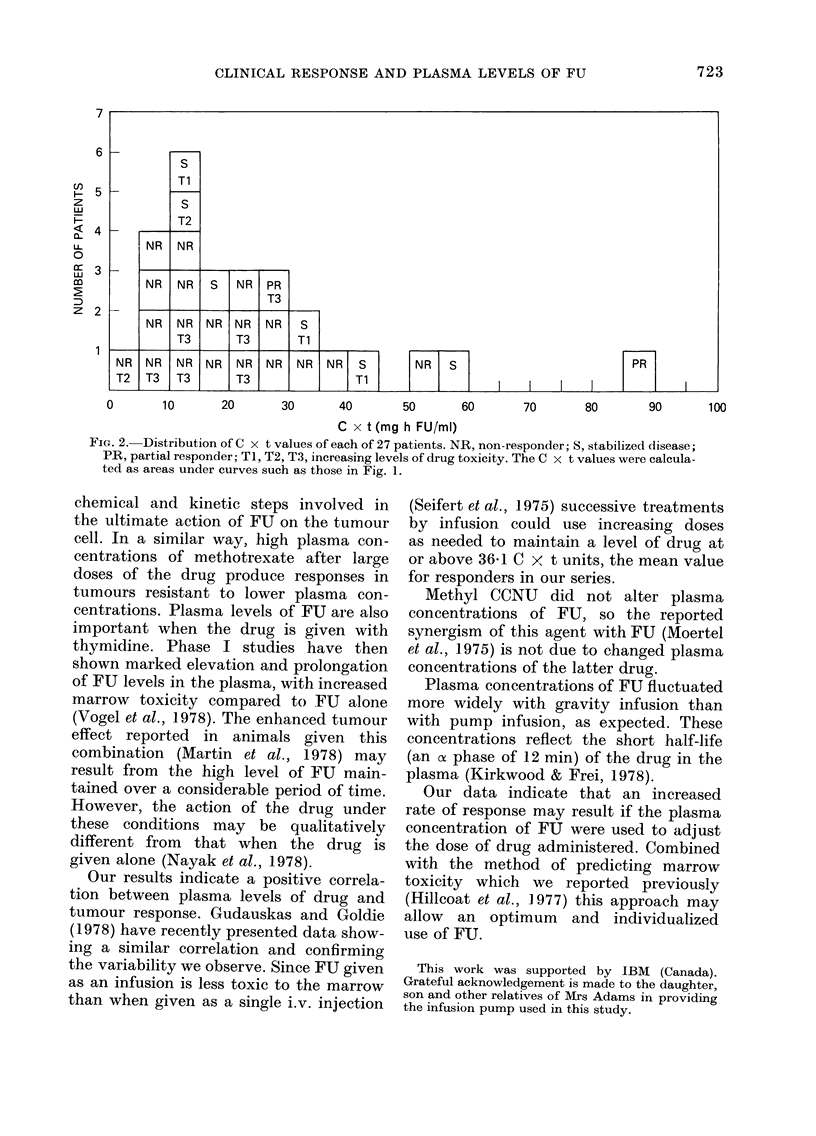

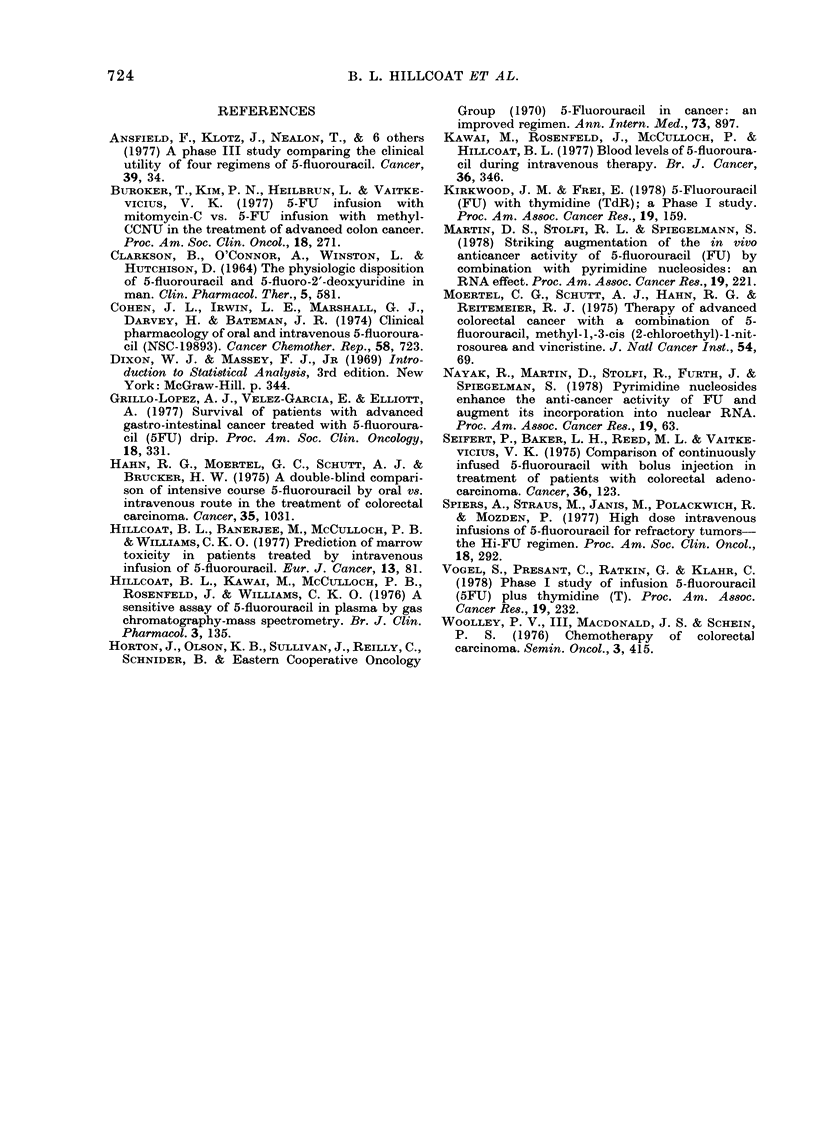


## References

[OCR_00787] CLARKSON B., O'CONNOR A., WINSTON L., HUTCHISON D. (1964). THE PHYSIOLOGIC DISPOSITION OF 5-FLUOROURACIL AND 5-FLUORO-2'-DEOXYURIDINE IN MAN.. Clin Pharmacol Ther.

[OCR_00793] Cohen J. L., Irwin L. E., Marshall G. J., Darvey H., Bateman J. R. (1974). Clinical pharmacology of oral and intravenous 5-fluorouracil (NSC-19893).. Cancer Chemother Rep.

[OCR_00810] Hahn R. G., Moertel C. G., Schutt A. J., Bruckner H. W. (1975). A double-blind comparison of intensive course 5-flourouracil by oral vs. intravenous route in the treatment of colorectal carcinoma.. Cancer.

[OCR_00817] Hillcoat B. L., Banerjee M., McCulloch P. B., Williams C. K. (1977). Prediction of marrow toxicity in patients treated by intravenous infusion of 5 fluorouracil.. Eur J Cancer.

[OCR_00822] Hillcoat B. L., Kawai M., McCulloch P. B., Rosenfeld J., Williams C. K. (1976). A sensitive assay of 5-fluorouracil in plasma by gas chromatography-mass spectrometry.. Br J Clin Pharmacol.

[OCR_00853] Moertel C. G., Schutt A. J., Hahn R. G., Reitemeier R. J. (1975). Therapy of advanced colorectal cancer with a combination of 5-fluorouracil, methyl-1,3-cis(2-chlorethyl)-1-nitrosourea, and vincristine.. J Natl Cancer Inst.

[OCR_00868] Seifert P., Baker L. H., Reed M. L., Vaitkevicius V. K. (1975). Comparison of continuously infused 5-fluorouracil with bolus injection in treatment of patients with colorectal adenocarcinoma.. Cancer.

[OCR_00888] Woolley P. V., Macdonald J. S., Schein P. S. (1976). Chemotherapy of colorectal carcinoma.. Semin Oncol.

